# *Staphylococcus aureus*—A Known Opponent against Host Defense Mechanisms and Vaccine Development—Do We Still Have a Chance to Win?

**DOI:** 10.3390/ijms23020948

**Published:** 2022-01-16

**Authors:** Urszula Wójcik-Bojek, Barbara Różalska, Beata Sadowska

**Affiliations:** Department of Immunology and Infectious Biology, Faculty of Biology and Environmental Protection, University of Lodz, Banacha 12/16, 90-237 Lodz, Poland; urszula.wojcik@biol.uni.lodz.pl (U.W.-B.); barbara.rozalska@biol.uni.lodz.pl (B.R.)

**Keywords:** *Staphylococcus aureus*, immune evasion, staphylococcal virulence factors, immune response, vaccines, molecular technologies, alternative therapies, antimicrobial resistance

## Abstract

The main purpose of this review is to present justification for the urgent need to implement specific prophylaxis of invasive *Staphylococcus aureus* infections. We emphasize the difficulties in achieving this goal due to numerous *S. aureus* virulence factors important for the process of infection and the remarkable ability of these bacteria to avoid host defense mechanisms. We precede these considerations with a brief overview of the global necessitiy to intensify the use of vaccines against other pathogens as well, particularly in light of an impasse in antibiotic therapy. Finally, we point out global trends in research into modern technologies used in the field of molecular microbiology to develop new vaccines. We focus on the vaccines designed to fight the infections caused by *S. aureus*, which are often resistant to the majority of available therapeutic options.

## 1. Introduction

Over many decades it has been established that, thanks to vaccines, efficient hygiene procedures and antibiotics, successful treatment and limitation of infectious diseases spread can be easily achieved. Indeed, the discovery and use of antibiotics, considered one of the greatest advances in medicine, was, in the beginning, a very promising direction. Unfortunately, their widespread and not always proper use in medicine, veterinary medicine and agriculture contributes to a growing phenomenon of antimicrobial resistance (AMR), mostly as a consequence of selection pressure, including the development of multiple drug resistance (MDR). As a result, treatment options for the most severe infections have gradually decreased over the years. The progress of microbial drug resistance was and is faster than the introduction of new drugs. Considering the above, it is strongly justified return greater attention once again to the improvement of specific (immune) prophylaxis, i.e., protective vaccinations introduced into medicine much earlier than antibiotic therapy. It is worth emphasizing that the efficacy of a vaccination is independent of the drug resistance profile of pathogens. Therefore, vaccines are a valuable weapon against microorganisms possessing an AMR/MDR phenotype. Moreover, the benefits of vaccination are much broader and include: (i) lowering the use of antimicrobial compounds, (ii) limiting the selection of resistant serotypes and (iii) reducing the infection rate of resistant strains in closely related species [1].

Ten years ago, the World Health Assembly endorsed the Global Vaccine Action Plan (GVAP) for 2011–2020. According to it, the indicated decade was expected to reduce the number of global deaths due to infectious diseases by ten times (25 million people). However, the analysis by the Strategic Advisory Group of Experts on Immunization (SAGE) of the World Health Organization (WHO) showed this goal was not met. It also suggested that due to a large backlog in this area, uneven contributions from individual countries and small financial outlays, it would not be possible to reach it in the near future. In addition, the COVID-19 pandemic situation has contributed significantly to the slowing down of the ability to achieve the GVAP targets. According to the experts, in order to extend the beneficial impact of vaccines and vaccination on global health, new vaccines for other diseases, as well as improved supply and delivery mechanisms, need to be developed [2].

Based on the information provided by the WHO, the list of infectious diseases/etiological agents targeted for vaccination and globally available comprises 16 items with the prevalence of viruses. Novel cell targets pending the urgent modification of the composition of current antibacterial vaccines or the development of an entirely new framework and formulations are needed. The list of vaccines that may be much more effective after improvement includes those against the following bacterial species: *Bordetella pertussis*, *Streptococcus pneumoniae*, *Neisseria meningitidis*, *Haemophilus influenzae* [3,4,5,6]. In addition to the vaccines already on this WHO list, there are important targets (viruses and bacteria) for new vaccines that are currently of little social, scientific, financial and manufacturing interest. Furthermore, the inventory of new “neglected” species of bacteria or those for which no vaccine has been developed, despite attempts, is also long and includes: Group A, B *Streptococcus* (*S. pyogenes*, *S. agalactiae*), invasive non-typhoid *Salmonella* (iNTS), *Shigella* sp., *Pseudomonas aeruginosa*, *Acinetobacter* sp. and *Staphylococcus aureus*. Although many different vaccine formulations have been proposed to prevent infections caused by some of the AMR pathogens (e.g., *S. aureus*, *Escherichia coli*, *Clostridioides difficile*), no successful phase III clinical trial data have been published yet. The multiple virulence mechanisms that a vaccine should target are among the numerous other possible reasons for the failure to develop vaccines against these pathogens [1,2,7,8].

In this review, the authors intend to provide the rationale for accelerating the research, pre-clinical and clinical trials needed to introduce a vaccine against *S. aureus* into immunotherapy. We highlight important reasons for this call to action, as well as the challenges. The background for these considerations is a description of the effective “abilities” of *S. aureus* to bypass/overcome/avoid the defense forces of the host, acting at particular stages of local or systemic infection. A presentation of the “history” of the proposed vaccines with the “*pros* and *cons*” arguments for selecting the individual *S. aureus* virulence factors as their components is also included. Finally, we discuss the trends in immune prophylaxis against *S. aureus* as well as several alternative strategies for combating this and other “difficult” bacteria.

## 2. *S. aureus* Infections as a Challenge to Vaccinology: Why Is It Important?

*Staphylococcus aureus* is a Gram-positive pathogenic bacterium that may be found on the skin and mucous membranes of humans and several animal species. *S. aureus* colonizes the nares or nasopharynx of about 30% of a population in an asymptomatic manner and becomes pathogenic after breaching epithelial barriers, making colonization an important risk factor [9,10,11,12]. These bacteria are the most common human pathogens, causing a wide variety of nosocomial and community-acquired infections. Typical staphylococcal infections range from minor skin and soft tissue infections, such as abscesses, furuncles and impetigo, to life threatening diseases, such as bacteremia, sepsis and toxic shock syndrome. In addition, *S. aureus* is a leading causative agent in surgical site infections, biomaterial-associated infections (e.g., catheters, artificial heart valves, bone and joint prostheses), cardiovascular infections, respiratory tract infections and food poisoning [13,14,15,16]. Epidemiological data show that *S. aureus* bacteremia leads to approximately 20,000 deaths a year in the USA, which accounts for more deaths than those from AIDS, tuberculosis and viral hepatitis combined together [17,18]. Furthermore, patients with risk factors (e.g., diabetics, immunocompromised, transplant recipients, oncological patients) are prone to developing severe staphylococcal infections and may require more frequent hospitalization [15,19,20,21]. In healthcare settings, *S. aureus* spreads rapidly, not only due to transmission from patients or medical staff, but also due to the ability of these microorganisms to survive on medical equipment and various hospital surfaces. The contamination of indwelling devices and implants represents a frequent route of infection because *S. aureus* easily adheres to abiotic surfaces, as well as to the matrix molecules that coat the devices after insertion. Staphylococci can also be transferred from the skin/mucous membranes of patient or healthcare personnel during surgery or other treatment and care procedures [21,22,23].

Treatment of staphylococcal infections is particularly problematic due to the constantly increasing acquisition of resistance genes and selection of antibiotic resistant *S. aureus* strains. Both community- and hospital-acquired methicillin-resistant *S. aureus* (CA-MRSA, HA-MRSA), representing multidrug-resistant strains (MDR), spread very quickly [16,24,25]. MRSA infections are associated with higher mortality and longer hospital stays than the infections caused by other pathogens, including methicillin-sensitive *S. aureus* (MSSA) [26,27,28]. Vancomycin was a last resort antibiotic for many years in such cases, but clinical isolates of vancomycin-resistant *S. aureus* (VRSA) have emerged and become a major public health concern [16,29]. Resistance to traditional beta-lactams and other available antibiotics limits the therapeutic options for treating these infections. The antibiotics currently approved for use in severe cases include glycopeptides, such as teicoplanin or dalbavancin, linezolid, tedizolid, daptomycin, tigecycline and cephalosporins of the fifth generation (ceftaroline, ceftobiprol) [16]. Finally, during the infection process, the staphylococci frequently form biofilms on both the inserted/implanted biomaterials and host tissues, which once established are difficult to eradicate and tend to recur.

A biofilm is a complex microbial community embedded in a self-made extracellular polymeric substance (EPS) that can be free-floating or attached to biotic or abiotic surfaces [30,31,32]. Bacteria in a biofilm exhibit different metabolism, gene transcription and protein production than during planktonic growth, which means completely new properties [33,34,35]. From the clinical point of view, one of the most important features is the higher tolerance of biofilms to environmental factors, which results in a weakened effect of antimicrobial drugs and impaired function of the host defense mechanisms [21,36,37]. The multifactorial tolerance/resistance of biofilms contributes to huge difficulties in combating biofilm-associated infections (BAI). Despite numerous studies regarding the new treatment methods of BAI, surgical excision of the infected tissue or removal of a colonized device is still the only viable solution in some cases [22].

### S. aureus Immune Evasion Strategies

The success of *S. aureus* as a pathogen depends on its ability to produce multiple virulence factors simultaneously and to regulate their expression quickly in response to environmental changes. As shown in Table 1, these factors allow staphylococci adhesion to the cell membranes or extracellular matrix proteins/glycoproteins as well as invasion of the host tissues. Then, they make them capable of avoiding immune response, which consequently leads to the spread of infection and serious damage throughout the body [38]. Selected mechanisms of this pathogen that interfere with the innate and adaptive immune system are discussed below.

One of the first barriers of a host’s innate immunity that *S. aureus* encounters during infection is the complement system—a proteolytic cascade of plasma proteins that can opsonize *S. aureus* cells, which promote their phagocytosis and killing by neutrophils (PMN—polymorphonuclear cells) and macrophages (M). Complement proteins may also lyse microbial cells directly by the activation and formation of a membrane attack complex (MAC) on the surface of the pathogen cell membrane [39,40]. The complement system can be activated through three activation routes (classical, lectin and alternative), and all of them result in the deposition of C3b on the staphylococcal surface. The C3a formed at the same time is an anaphylatoxin with a range of functions in inflammatory response, including macrophage and T cell activation as well as chemotaxis.

*S. aureus* produces multiple proteins that interfere with complement activation [39,43,44]. The most versatile one, SCIN, blocks all three pathways by inhibiting C3 convertases and thereby decreasing C3b deposition, C5 convertase formation and the release of chemoattractant C5a [43,45]. Another important factor, Efb, binds both C3 and fibrinogen, thus covering bacteria with a thick layer of fibrinogen that shields the C3b and antibodies from recognition by phagocytic receptors [46]. Several cell wall-associated proteins also inhibit complement activation—Cna binds to C1q and, therefore, inhibits the classical pathway [47], while SdrE recruits factor H to the surface of staphylococci, leading to inhibition of the alternative pathway [48]. In addition, *S. aureus* secretes proteases that degrade the complement proteins, including Aur and Scp A [49,50]. Together, these evasion mechanisms lead to a reduction in the complement-aided phagocytosis of bacteria and inhibition of MAC formation.

Phagocytes are the most important components of innate immunity that migrate to the site of infection and represent another line of host defense against pathogens. Neutrophils are abundantly present in blood, while macrophages reside in tissues or derive from circulating monocytes, but both play a key role in recognizing, engulfing and killing staphylococci [40,51,52,53]. Most clinical strains of *S. aureus* produce a polysaccharide capsule that protects cell wall proteins from being recognized by the phagocyte receptors [39,54,55]. Coagulase and ClfA also play an important role in avoiding phagocytosis—coagulase activates prothrombin to polymerize fibrinogen (Fg), whereas ClfA allows staphylococci to bind Fg. Both these events lead to fibrin-/fibrinogen-bacteria clump formation [56,57,58]. The formation of a fibrin network or fibrinogen clumps protects the bacteria from neutrophil clearance [40]. However, if the phagocytes manage to ingest *S. aureus* cells, the real attack begins inside the phagosome, where the bacteria are subjected to high levels of reactive oxygen species (ROS), enzymes and antimicrobial peptides (AMPs) [52]. *S. aureus* uses multiple strategies to interfere with both oxygen-dependent and -independent bactericidal mechanisms. One of them is staphyloxanthin—a carotenoid pigment that scavenges free radicals and gave *S. aureus* its name [59,60]. Additionally, this pathogen produces several enzymes that prevent ROS formation, such as superoxide dismutase (SOD), which converts superoxide radicals into hydrogen peroxide, and catalase, which further degrades it into water and oxygen [60,61,62]. Staphylococci reduce the negative charge of their membranes and cell walls through the incorporation of D-alanine into teichoic acid and L-lysine into membrane phosphatidylglycerol, in order to protect themselves against positively charged antimicrobial peptides (AMPs) [63]. They also secrete factors that degrade cathelicidin LL-37 (Aur) and α-defensin (SAK), as well as inhibit neutrophil serine proteases (Eap) [38,40,63]. Moreover, *S. aureus* can persist, and even replicate in mature phagolysosome, due to adaptation to an acidic microenvironment. The influence of stress factors on staphylococci results in the formation of slow-growing subpopulations of bacteria—small colony variants (SCVs), characterized by a high resistance to antibiotics and the host defense mechanisms. It has also been suggested that during continuous contact with staphylococci, the infected macrophages eventually lose their ability to kill this pathogen, so *S. aureus* can multiply and then trigger cell death to disseminate throughout the body [64,65,66]. Finally, neutrophils and macrophages form extracellular traps (ETs), which often lead to their own death (ETosis). These traps consist of DNA, histones, AMPs, and enzymes and serve to stop spreading the pathogens and kill them, but *S. aureus* can easily escape from ETs using the nucleases and proteases [67].

*S. aureus* secretes numerous toxins that kill immune cells by disrupting the cell membrane (necrosis) or by inducing programmed cell death (apoptosis/autophagy/pyroptosis) to protect itself against phagocytosis, establish infection and disseminate in the host [68]. The most important pore-forming toxins include α-hemolysin (Hla), Panton–Valentine leukocidin (PVL) and phenol-soluble modulins (PSMs). Hla is a major toxin of *S. aureus* in terms of contribution to the pathogenesis that is manifested in the diversity of its functions. Some of these include the disruption of epithelial and endothelial barriers, induction of inflammatory response and lysis or apoptosis of immunocompetent cells [58,68,69,70]. Alpha toxin lyses a wide range of human cell types (e.g., erythrocytes, platelets and leukocytes, including T cells), while PVL targets mainly neutrophils, monocytes and macrophages. Although PVL is produced by only 2–3% of *S. aureus* isolates, it is found in most community-acquired MRSA strains, especially those responsible for pneumonia [68,71,72]. Another class of toxins are PSMs, or amphipathic peptides with considerable cytolytic activity against many types of eukaryotic cells, e.g., bone cells, endothelial and epithelial cells, monocytes, erythrocytes and mostly against neutrophils [68,73]. Their function is inhibited by serum lipoproteins; thus, they reach a high concentration inside the condensed phagosomes [74]. PSMs are associated with enhanced virulence in CA-MRSA skin infections [75].

*S. aureus* is successful in avoiding not only the innate immune system but also in interfering with the development of an antigen-specific response. For this purpose, it uses, for instance, surface-bound protein SpA that can bind the immunoglobulins, TNFα receptor 1, von Willebrand factor and C1qR component of the complement. SpA is expressed by most clinical isolates [76,77,78]. This protein consists of five immunoglobulin-binding domains capable of binding both the Fc regions of IgG and IgM, thus blocking opsonophagocytosis and complement activation, as well as the Fab domain of some immunoglobulins, including variable heavy 3 (VH3) of the B cell receptors, which leads to the activation and clonal expansion of B cells [77,79]. As a result, the B cells recognize, almost exclusively, protein A and the host response against other staphylococcal virulence factors is then limited [78]. Moreover, *S. aureus* also manipulates the T cell response by staphylococcal superantigens (e.g., TSST-1, enterotoxins) that non-specifically activate T cells by cross-linking the major histocompatibility complex of class II and the T cell receptor (TCR). This causes a massive activation of T cells and the release of proinflammatory cytokines (cytokine storm), leading to life-threatening toxic shock syndrome and multi-organ dysfunction [80,81,82].

In summary, *S. aureus* has evolved multiple strategies to avoid the host immune system response, which contributes to difficulties in both treating staphylococcal infections and developing preventive options such as vaccine or immune therapy. However, attempts are constantly being made to win this fight.

## 3. Past and Present in Active and Passive Immunotherapy of Staphylococcal Infections

*S. aureus* is one of the best-known bacteria in terms of virulence factors and their participation in pathogenesis. A wide repertoire of staphylococcal adhesive molecules, toxins and enzymes seems to be an excellent starting point to choose proper antigens for vaccine development. Conversely, such diversity allows these bacteria to avoid the activity of specific antibodies by replacing one virulence factor with another during a regular life cycle or by using toxins and enzymes to dampen the immune response. For example, many staphylococcal adhesive molecules from MSCRAMMs are capable of interacting with the same host extracellular matrix proteins (ECM), which is why blocking just one of them does not limit *S. aureus* adhesion and biofilm formation [38,83]. Moreover, as described in Section 2, *S. aureus* cytotoxins, such as hemolysins, leukocidins and PSMs, effectively damage immune cells, including neutrophils, monocytes, macrophages, dendritic cells (DC) and lymphocytes. Given the adaptive immune response, the elimination of B lymphocytes through the formation of pores in cell membranes or through the activation of programmed cell death seems to be of particular importance [38,51,84]. It is also worth mentioning yet againthe ability of *S. aureus* surface protein A (SpA) to bind the Fc domains of IgG and IgM, which blocks their interaction with the immune cells and the activation of the complement [77,79]. Furthermore, α-toxin and other staphylococcal immunogenic antigens secreted into host tissues very often interact with specific B lymphocytes and antibodies away from the site of *S. aureus*, causing cell inactivation without co-stimulating a signal from the T lymphocytes [84]. Taking all this into account, the creation of an effective anti-staphylococcal vaccine is quite difficult, similar to the usage of specific antibodies in passive immunotherapy.

The first vaccine programs targeting single *S. aureus* virulence factors ended in failure. The vaccine containing iron surface determinant B (IsdB), necessary for iron acquisition, did not pass the safety tests. In the phase IIb/III clinical trials the V710 vaccine increased the mortality of immunized humans because of *S. aureus* infections. Nishitani et al. [85] proved with a mouse model that the antibodies specific to IsdB facilitated bacterial entry into the leukocytes, and their dissemination was the cause of increased host susceptibility to sepsis following an *S. aureus* local infection. Thus, anti-IsdB antibodies may be, at least in part, the cause of the V710 vaccine failure [85]. The StaphVax vaccine, comprising two predominant staphylococcal capsular polysaccharide (CP) serotypes (CP5 and CP8) conjugated to the recombinant *Pseudomonas aeruginosa* exoprotein A, was one of the first bivalent preparations developed; it achieved phase III efficacy studies and failed [7,86]. The formula: capsular polysaccharide with an immunogenic protein carrier that has been successfully used in the vaccines against such pathogens as *Neisseria meningitidis* (CP conjugated to the nontoxic recombinant variant of tetanus toxin) or *Streptococcus pneumoniae* (CP conjugated to the nontoxic recombinant variant of diphtheria toxin—CRM_197_ or surface lipoprotein D of *Haemophilus influenzae*). This was found to be safe but ineffective in the case of staphylococci [86,87,88]. Therefore, multivalent anti-staphylococcal vaccines containing a few different antigens targeting multiple virulence mechanisms started to be developed. The CP5 and CP8 (each conjugated to CRM_197_), a recombinant clumping factor A (rClfA) and, additionally, a recombinant lipoprotein rP305A obtained from a manganese transporter C (MntC) were used as the target antigens in the 3-antigen (SA3Ag) and 4-antigen *S. aureus* vaccine (SA4Ag), respectively [86,89,90,91]. The use of all these antigens is fully justified, not only because of their immunogenicity. As stated in the previous section, CP allows the bacteria to avoid opsonophagocytosis and elimination by the host innate immune system. ClfA, as one the most potent adhesive molecules, participates in microbial adhesion, biofilm formation and interaction with the host cells/tissues, as well as renders the bacteria coated with ECM, invisible to the immune system. MntC is a surface molecule responsible for the acquisition of manganese as a cofactor of many enzymes essential for bacterial metabolism, signal transduction, cell division and the host immune evasion [86,92]. Moreover, all of these antigens are over-produced mainly at the beginning of an infection to stabilize colonization, multiplication, possible subsequent biofilm formation and, finally, dissemination [86]. Phase I and II clinical trials in healthy adults demonstrated that the SA3Ag and SA4Ag offered acceptable safety and tolerability. Both vaccines also induced a rapid and robust immune response leading to a generation of functional specific antibodies against all antigens used, observed even through 36 months post-vaccination [89,90,91]. Because *S. aureus* surgical site infections (SSIs) followed by systemic infections (bacteremia and sepsis) are the most common and life-threatening complications of orthopedic surgeries, a search for their prevention seems particularly important. Thereby, phase IIb/III clinical trials testing the safety and efficacy of the SA4Ag in adults undergoing elective open posterior multilevel instrumented spinal fusion surgery (STRIVE study—*STaphylococcus aureus* suRgical Inpatient Vaccine Efficacy) were conducted. The patients who were vaccinated prior to surgery and monitored afterwards demonstrated both the safety and efficacy of the SA4Ag. Thus, similar benefits of vaccination (the prevention of postoperative *S. aureus* infections) for the patients requiring other orthopedic surgeries were suggested [86]. In 2019, the SA4Ag was described as the most advanced anti-staphylococcal vaccine; however, the studies are still in progress.

The main goal is to stop *S. aureus* infection as early as possible, to disallow the formation a resistant to immune intervention biofilm and/or to prevent later complications, such as cytokine storm and toxic shock syndrome. The current paradigm for vaccine development is targeting multiple staphylococcal virulence factors, considering both the surface antigens and secreted biologically active substances. Thus *S. aureus* adhesins and other surface proteins will generate opsonic antibodies, while non-toxic forms of toxins and superantigens will stimulate the production of neutralizing antibodies and activate effector immune cells [93,94]. An example of such a complex preparation is the *S. aureus* toxoid vaccine, containing modified bi-component pore-forming toxins: mutants of the S and F subunits of PVL (LukS_mut9_ and LukF_mut1_) and a double mutant of alpha hemolysin (Hla_H35L/H48L_), as well as the fusion toxoid TBA_225_ of superantigens (SEA, SEB and TSST-1). The toxoid vaccine was tested in the non-human primate model (rhesus macaques) and described as safe, well tolerated and immunogenic. The post-vaccination titers of the specific IgG with neutralizing activity were many times higher than the baseline values (from 5 to 190 times depending on the antigen). Moreover, the population of antigen-specific T cells of the Th1 and Th17 phenotype, as well as the production of some T cell cytokines (e.g., TNF, IFN-γ, IL-2, IL-17A), increased in response to ex vivo re-stimulation of peripheral blood mononuclear cells with the vaccine. It is suggested that such a T cell response may, via feedback, activate the innate immune cells that enhance phagocytosis and bacteria elimination [94].

The B cell-mediated immune response to *S. aureus* infection triggers the production of specific antibodies against many staphylococcal antigens, including cell wall components, such as peptidoglycan and lipoteichoic acid, capsular polysaccharides, pore forming toxins or superantigens [93]. Thus, on the one hand, the idea of using passive immunization as a form of antistaphylococcal therapy seems quite reasonable. On the other hand, the question is why do recurrent *S. aureus* infections happen despite the production of specific anti-staphylococcal antibodies? Regardless, many clinical trials on passive immunization with monoclonal or polyclonal antibodies against *S. aureus* antigens (e.g., ClfA, CP5 and CP8, PNAG, Hla, HlgAB) were carried out [95]. Rupp et al. [96] presented the results of a phase II randomized, multicenter, double-blind, placebo-controlled trial of polyclonal anti-*S. aureus* CP5/CP8 antibodies (Altastaph) in patients with staphylococcal bacteremia. A shortened median time of fever, as well as hospitalization, for Altastaph recipients, in comparison to the patients from the placebo group, was demonstrated. However, mortality was similar or even greater in the tested group than in control [96]. The monoclonal antibodies against LTA (Pagibaximab) or PNAG also failed in phase III or phase IIa trials, respectively [95]. Thus, passive immunization with opsonic antibodies targeting *S. aureus* cell wall components or envelope polymers cannot be recognized as a strategy solving the problem of staphylococcal infection treatment. Similarly, as with vaccines, the current trend in passive immunization focuses on the antibodies against secretory biologically active staphylococcal products, including pore-forming toxins. For example, human monoclonal anti-*S. aureus* alpha toxin antibodies, known as MEDI4893, undergo clinical trials aimed at the prevention of *S. aureus* pneumonia [95,97]. This strategy will be discussed in Section 4.

As specific antibodies seem not to be fully sufficient for human protection against *S. aureus* infections, greater attention has recently shifted to immunotherapy promoting innate immune response. The modulation of selected cytokine production and the phenotypic reprogramming of innate immune cells to develop innate immune memory as a key element of anti-infective protection are seriously considered [98,99,100,101]. Among cytokines, IL-17 seems to be critically important for the host immune response against many types of pathogens, including *S. aureus*. It activates CXC chemokine production (e.g., CXCL8, CXCL1, CXCL2) and, consequently, the recruitment of neutrophils to the site of infection and microorganism elimination [101,102]. Moreover, IL-17A promotes the expression of host defense peptides in the skin (in vivo studies on murine model), which are an important part of innate anti-staphylococcal response [102]. IL-17 is produced by Th17, and also by innate immune cells, such as γδT cells, known to be recruited to wounds infected by *S. aureus*, and, thus, a primary source of this interleukin. Further, invariant natural killer T cells (iNKTs) and lymphoid tissue inducer (LTi)-like cells are capable of producing IL-17 [101,102,103]. Therefore, IL-17 and IL-17-producing cells are proposed to be the targets for novel antistaphylococcal immunotherapeutic strategies.

In the context of the importance of innate immune response and memory, the effect of prior contact of mice with *S. aureus* (priming) on the course of staphylococcal skin infection was studied [98,99,103]. Much smaller skin abscesses with a lower number of staphylococci were formed in the primed mice than in the naive ones. It was accompanied by a significantly increased infiltration of innate immune cells, such as PMN, M (mainly polarized to M1), DC and natural killer cells (NK) into the infection sites of the primed mice. Priming also induced greater production of some cytokines (IL-17, IL-6, IL-22, IFN-γ, MIG—monokine induced by IFN-γ) and antimicrobial peptides (CRAMP and mβD-3) [98,99]. In a mouse model of *S. aureus* skin reinfection, Dillen et al. [103] showed that primary contact with *S. aureus* promoted cloning of γδT cells, producing TNF and IFN-γ, which boosted the host defense against subsequent staphylococcal infection. These observations clearly indicate that the contact with *S. aureus* triggers the development of some innate memory. Therefore, our understanding of the mechanisms of innate immune response stimulation and, in the future, their modulation to develop the “trained immunity” hold great promise for the prevention and treatment of *S. aureus* infections.

## 4. The Prospects of Immune Prophylaxis Trends against *S. aureus*

As discussed in detail above, achieving the GVAP goals mentioned in Section 1 will not be easy in the near future, at least for some infections. Despite huge progress in research on the bacterial species with multiple virulence and immune evasion factors, it is still difficult to find tsatisfactory tools for ensuring victory. During the development of the vaccines tested thus far, many classic methods and several new technological solutions have been used, and the expected results have yet to be achieved. Although some potential anti-*S. aureus* vaccines showed protective efficacy in preclinical or early clinical trials, none have been approved for use in humans to date [7,104]. Most researchers reasonably believe that we still need to learn more about new technologies in vaccine formulation and performance in order to overcome the limitations of the previous versions. The new approaches, such as reverse vaccinology, novel adjuvants, structural vaccinology, bioconjugates and rationally designed bacterial outer membrane vesicles (OMVs), seem promising. These lines of research, together with progress in polysaccharide conjugation techniques and antigen in silico design, could be the center of future vaccine development (VRD). As the discussion of these approaches exceeds the assumed scope of this review, we recommend several interesting links for publications on this topic [105,106,107,108,109,110,111].

In the final part of this review, we want to highlight only a few promising research directions [112]. An interesting perspective on vaccine “strategy” against *S. aureus* was proposed by Klimka et al. [113]. They suggested narrowing the composition of the vaccine to small epitopes of coproporphyrinogen III oxidase (CgoX) and triose phosphate isomerase (TPI). These epitopes fulfill essential housekeeping functions in heme synthesis and glycolysis, respectively. Two types of monoclonal antibodies (mAb), raised against CgoX and TPI, provided efficient protection against *S. aureus* infection when used for passive immunization. According to the authors, such a strategy will ensure spectacular precision of the vaccine. Their encouraging observation that in over 97% of the more than 35,000 investigated clinical strains of *S. aureus* these epitopes remain unchanged, and thus this vaccine candidate will have a broad effect, is very interesting. Therefore, “epitope-focused immunization” represents a new trend in vaccine development based on the preparation of mAb with narrow specificity. Monoclonal antibodies are now an integral part of the “reverse vaccinology 2.0” concept, where Abs are used to distinguish protective from non-protective epitopes and to support immune-focused antigen design. This strategy is anticipated to improve its immunogenic precision level, resulting in a vaccine with a greater efficacy and safety profile [105,114].

Alternatively, in addition to active immunization, the use of novel antibody-based passive immunization strategies, as described in Section 3, also might offer some hope. One current (though not quite new) concept is targeting the neutralization of *S. aureus* toxins. Mentioned in a previous section, the MEDI4893—human monoclonal antibodies specific to *S. aureus* α-toxin (Hla)—have been dedicated for mechanically ventilated patients to prevent staphylococcal pneumonia. Targeting Hla as a highly conserved and pivotal *S. aureus* virulence factor participating in tissue disruption, programmed cell death, immune response dysregulation and bacterial dissemination seems to be the key to the success of immune prophylaxis. Moreover, the MEDI4893 contain a triple-amino-acid substitution in the Fc region to extend their serum half-life. A phase I clinical trial confirmed their safety and tolerability, as well as their neutralizing potential (ex vivo study) [97]. Although future studies are necessary for such a strategy to be implemented, Miller et al. [93] emphasize that *S. aureus* toxins, especially superantigens and pore-forming toxins that disrupt the host innate and adaptive immune responses are important targets in protective immunity. Therefore, naturally-generated or vaccine-induced antibodies specific to *S. aureus* toxins are, or should be, associated with improved clinical outcomes. However, there are some limitations concerning their efficacy: (i) genetic variability among humans in terms of response to the toxins, (ii) variable expression of the plethora of toxins by *S. aureus* strains, (iii) variable level of antitoxin antibodies production and (iv) probably different host responses to the toxins depending on anatomical sites of infection [93,115]. Thus, this direction of research also needs to be intensified with the use of more modern techniques and tools than those currently available.

## 5. Conclusions

As mentioned in Section 1, in 2011, as many as 25,000 deaths in the European Union (EU) were attributed to antimicrobial resistance (AMR). It is calculated that without policies and actions to stop the spread of AMR, the number of deaths in Europe could grow up to 390,000 every year by 2050. Evidence has shown that existing vaccines have a positive impact on the reduction in AMR; therefore, the recommended strategies include reasonable use of antimicrobials and greater infection control measures [116,117]. However, it is obvious that National Immunization Programs and vaccination planning in the EU should be integrated to meet the expectations. A clear vision for vaccine research and development (VRD) is needed in Europe to continue leading to the invention of next-generation vaccines. The Innovation Partnership for a Roadmap on Vaccines in Europe (IPROVE) has been launched. It is a collaboration among leading vaccine experts to develop a roadmap that outlines how Europe can best invest in the science and technology essential for vaccine innovation [118].

It is well accepted that to maximize the impact of vaccines in reducing the emergence of antimicrobial resistance, most of the population that is at risk of infection should be vaccinated, including all countries where the antimicrobial-resistant pathogens are endemic. Unfortunately, for most of the key AMR pathogens, vaccines are either not yet available or widespread social acceptance is unsatisfactory. More must be done to understand and address the reasons for vaccination hesitancy of the general public and healthcare professionals. The WHO and other official Health Services Reports say that while it is essential to develop new antibiotics, there is no guarantee that enough will be discovered to tackle the threat of antimicrobial resistance in the long term. Thus, other approaches (new vaccines) to prevent and treat the infections are needed. It is also strongly recommended that in the short term, the use of existing vaccines needs to be increased in both humans and animals [106,107,108,109,110,111,117].

As the world rightly focuses on limiting the spread of COVID-19, the current pandemic situation has exposed our vulnerability to the infections for which there are no effective vaccines or treatments [105,106]. An integrated strategy that includes multitargeted vaccines together with modern diagnostic tools, novel antibiotics, monoclonal antibodies, microbiota modulation, as well as the use of antimicrobial peptides, bacteriocins, plant-derived products and bacteriophages are required to combat AMR effectively [119,120,121,122,123,124]. The still-developing research trend concerning interference with the expression of pathogen virulence factors at the molecular and submolecular levels is also very important. It is assumed that the achievements in this field may turn out to be a key tool in the modern prevention and therapy of AMR-caused infections in the near future.

## Figures and Tables

**Table 1:** The most important virulence factors of *S. aureus* and their targets during infection [38,41,42].

Type of Virulence Factor	Name	Target	Effect
Cell wall-associated factors	Cell wall components—peptidoglycan, teichoic acid, lipoteichoic acid	Immune cells, other tissues	Stimulate immune cell activation and inflammatory response; participate in adhesion and biofilm formation
	Staphylococcal protein A (SpA)	IgG, IgM, complement	Binds Fc region of IgG and IgM, thus inhibiting opsonization and phagocytosis; activates B cells
	Fibronectin-binding proteins (FnBPA, FnBPB)	Fibronectin, fibrinogen, elastin, plasminogen, keratin, complement	Binding to extracellular matrix proteins (ECM), enable adhesion to host tissues and biomaterials; limit phagocytosis and complement activation
	Collagen-binding protein (Cna)	Cartilage and collagen-rich tissues, complement	Binding cartilage and collagen, enables adhesion to host tissues; inhibits complement activation
	Clumping factors (ClfA, ClfB)	Fibrinogen, blood platelets, complement (ClfA), cytokeratin 10 (ClfB)	Binding to fibrinogen, enables adhesion to host tissues; inhibit complement preventing opsonization and phagocytosis; activate platelets
	Serine-aspartate repeat protein E (SdrE)	Complement	Inhibits complement preventing opsonization and phagocytosis
	Iron-regulated surface determinant proteins (IsdA, IsdB)	Heme-iron	Heme uptake and iron acquisition contribute to increased pathogenesis, tissue invasion and abscess formation
	Polysaccharide intercellular adhesion/polymeric N-acetyl-glucosamine (PIA/PNAG)	Staphylococcal cells, mucous membranes, other tissues, abiotic surfaces	Participates in bacterial aggregation, adhesion and biofilm formation (major component of biofilm matrix); reduces phagocytosis
	Capsular polysaccharides	Mucous membranes, other tissues, abiotic surfaces	Reduce phagocytosis; increase the efficiency of colonization and durability on the surface of mucous membranes or biomaterials
Enzymes	Catalase	Hydrogen peroxide	Catalyzes breakdown of hydrogen peroxide into water and oxygen, preventing oxidative stress
	Coagulase	Prothrombin	Reacts with prothrombin, allowing fibrinogen polymerization and clot formation, thus reducing phagocytosis
	Staphylokinase (SAK)	Plasminogen	Converts plasminogen to active serine protease plasmin, which promotes degradation of ECM, complement and IgG
	Lipases	Lipids of cell membranes and components of sebum	Decompose lipids, which allows spreading of staphylococci
	Nucleases	Nucleic acids	Degrade nucleic acids, thereby releasing them from extracellular traps (ETs)
	Proteases, e.g., serine protease V8 (SspA), staphopain A (Scp A) and B (SspB), aureolysin (Aur)	ECM proteins, complement, mucins, pulmonary surfactant	Degrade ECM proteins, mucins and pulmonary surfactant, which allow staphylococcal spread in the host tissues; inhibit chemotaxis and phagocytosis by proteolysis of immune cell receptors; degrade complement preventing opsonization and lysis of bacteria; degrade antimicrobial peptides
	Superoxide dismutases	Superoxide	Convert superoxide to hydrogen peroxide and oxygen, thereby preventing oxidative stress
Toxins	Hemolysins (alpha, beta, gamma, delta)	Erythrocytes, platelets, leukocytes	Cause lysis of red blood cells, platelets, leukocytes—evading of host immune response; bacterial spreading
	Enterotoxins	Enterocytes, lymphocytes T	Cause diarrhea; after translocation into blood, activate lymphocytes T leading to cytokine storm
	Exfoliative toxins	Desmosomes between keratinocytes	Cleave the granular layer of the epidermis by damaging desmosomes (staphylococcal scalded skin syndrome)
	Panton-–Valentine leukocidin (PVL)	Neutrophils, monocytes, macrophages	Causes lysis of neutrophils, monocytes, macrophages—avoiding innate immune response; development of necrotic changes
	Toxic shock syndrome toxin 1 (TSST-1)	Lymphocytes T	Activates lymphocytes T, which causes massive production of cytokines and leads to toxic shock syndrome
Other secreted proteins	Chemotaxis inhibitory protein of *Staphylococcus* (CHIPS)	Neutrophils	Binds to cell receptors (FPR1 and C5aR) inhibiting neutrophils chemotaxis, thereby preventing phagocytosis
	Staphylococcal complement inhibitor (SCIN)	Complement (C4, C3b)	Inhibits complement activation, thus preventing bacterial lysis, opsonization and phagocytosis
	SSL-5	Neutrophils, platelets	Binds to cell receptors (PSGL-1 and GPCRs) inhibiting neutrophil diapedesis and activation; activates platelets (aggregate formation)
	SSL-7	IgA, complement (C5)	Binds Fc region of IgA and complement protein C5, thus blocking antibodies and inhibiting complement activation
	Extracellular fibrinogen-binding protein (Efb)	Fibrinogen, blood platelets, complement	Binds fibrinogen enabling adhesion and aggregation: interferes with platelet aggregation; inhibits complement activation
	Extracellular adherence protein (Eap)	ICAM-1	Binds ICAM-1 inhibiting neutrophil rolling and migration (diapedesis)
